# Detection of mammaglobin mRNA in peripheral blood is associated with high grade breast cancer: Interim results of a prospective cohort study

**DOI:** 10.1186/1471-2407-8-55

**Published:** 2008-02-20

**Authors:** Kaidi Mikhitarian, Renee Hebert Martin, Megan Baker Ruppel, William E Gillanders, Rana Hoda, Del H Schutte, Kathi Callahan, Michael Mitas, David J Cole

**Affiliations:** 1 Department of Surgery, Medical University of South Carolina, 171 Ashley Ave, Charleston, SC 29425, USA; 2 Department of Biostatistics, Bioinformatics and Epidemiology, Medical University of South Carolina, 135 Cannon Street, Charleston, SC 29425, USA; 3 Department of Pathology and Laboratory Medicine, Medical University of South Carolina, 171 Ashley Ave, Charleston, SC 29425, USA; 4 Department of Orthopaedic Surgery, Medical University of South Carolina, 171 Ashley Ave, Charleston, SC 29425, USA; 5 Pathology and Anatomical Sciences, University of Missouri – Columbia, One Hospital Drive, Columbia, MO 65212, USA; 6 Section of Endocrine and Oncologic Surgery, Washington University School of Medicine, 660 S. Euclid, Saint Louis, MO 63110, USA

## Abstract

**Background:**

We sought to examine the detection rate of cancer cells in peripheral blood (PBL) and in bone marrow (BM) using an established 7-gene marker panel and evaluated whether there were any definable associations of any individual gene with traditional predictors of prognosis.

**Methods:**

Patients with T1-T3 primary breast cancer were enrolled into a prospective, multi-institutional cohort study. In this interim analysis 215 PBL and 177 BM samples were analyzed by multimarker, real-time RT-PCR analysis designed to detect circulating and disseminated breast cancer cells.

**Results:**

At a threshold of three standard deviations from the mean expression level of normal controls, 63% (136/215) of PBL and 11% (19/177) of BM samples were positive for at least one cancer-associated marker. Marker positivity in PBL demonstrated a statistically significant association with grade II-III (vs. grade I; p = 0.0083). Overexpression of the mammaglobin (*mam*) gene alone had a statistically significant association with high tumor grade (p = 0.0315), and showed a trend towards ER-negative tumors and a high risk category. There was no association between marker positivity in PBL and the pathologic (H&E) and/or molecular (RT-PCR) status of the axillary lymph nodes (ALN).

**Conclusion:**

This study suggests that molecular detection of circulating cancer cells in PBL detected by RT-PCR is associated with high tumor grade and specifically that overexpression of the *mam *gene in PBL may be a poor prognostic indicator. There was no statistically significant association between overexpression of cancer-associated genes in PBL and ALN status, supporting the concept of two potentially separate metastatic pathways.

## Background

There is a significant amount of ongoing work aimed at defining the role of circulating tumor cells (CTC) in peripheral blood (PBL) and disseminated tumor cells (DTC) in bone marrow (BM) of breast cancer patients. However, due to a variety of available tumor cell detection methods and use of different gene-markers, recently published studies show a wide range of results that are often contradictory and difficult to compare to one another. The main tumor cell detection methods have been immunocytochemistry (ICC) with cytokeratin-specific antibodies [1-11] and RT-PCR analysis based on overexpression of cancer-associated gene-markers [4,6,12-29]. PCR methodology for detection of breast cancer has most frequently employed mammaglobin (*mam*) and cytokeratin 19 (*CK19*) genes. Some studies have also used a new CellSearch System technology that employs immunomagnetic separation of epithelial cells based upon expression of cytokeratins or *EpCAM *and visualization of the tumor cells by immunoflorescent microscopy [30].

Our laboratory has extensive experience in detection of cancer cells using multi-marker real-time RT-PCR methodology [31-35]. To address the clinical relevance of molecular detection of occult breast cancer, we initiated a multi-institutional prospective cohort study. The primary objective of the study was to determine whether the molecular detection of occult breast cancer by multi-marker real-time RT-PCR in patients with pathology-negative axillary lymph nodes (ALN) is a clinically relevant predictor of disease recurrence. An interim analysis of 489 patients enrolled in the study showed a statistically significant association between molecular detection of occult breast cancer in the ALN and traditional predictors of poor prognosis in subjects with pathology-negative ALN [33]. In addition, in a separate publication we show that the sensitivity of sentinel lymph node (SLN) analysis to predict pathologic status of ALN was significantly increased by the addition of molecular analysis [34].

There are several cancer-associated gene markers used in the detection of breast cancer cells. Based on the heterogenous nature of the breast cancer, the multi-marker panel approach has shown to increase the sensitivity of molecular assay to detect the presence of disseminated cancer cells. However, the prognostic value of each individual marker is not known and therefore the ultimate goal would be to identify genes that are capable of differentiating patients with poor prognosis from the patients with a more favorable prognosis. Having a tool to recognize the subset of patients with unfavorable molecular characteristics could potentially translate into a better clinical outcome. In this interim analysis we examine the detection rate of cancer cells in PBL and in BM using an established 7-gene marker panel and evaluated whether there were any definable associations of any individual gene with the traditional predictors of prognosis.

## Methods

### MIMS Trial Study Design

A prospective cohort study design was adopted where, upon recruitment, eligible participants with Stage I, IIa, or IIb breast cancer were requested to consent to tissue sampling from axillary lymph nodes (ALN), sentinel nodes (SLN), bone marrow (BM), and peripheral blood (PBL). Tissue sampling was accomplished at the time of surgical intervention. The study was carried out in compliance with the Helsinki Declaration ethical principles in medical research involving human subjects. All specimens were collected through the Medical University of South Carolina Institutional Review Board for Human Research approved protocols (HR 9551, HR 8374, HR 8903, HR 8432). Informed consent was obtained in accordance with each participating center's Institutional Review Board guidelines. The design, enrollment criteria, tissue acquisition protocols, and determination of gene expression values for patients enrolled in the MIMS trial are described in more detail in a separate publication [33]. The current study focuses on the subset of 215 patients with PBL samples and the subset of 177 patients with BM samples. Real-time RT-PCR analyses for cancer-associated genes were performed on all specimens at the Central Molecular Diagnostics Laboratory at the Medical University of South Carolina (MUSC). The Clinical Innovation Group (TCIG, Charleston, SC) (later known as the Data Coordination Unit (DCU) in the Department of Biostatistics, Bioinformatics and Epidemiology at MUSC) served as the coordinating center, and all study data were collected, processed and analyzed at this central facility.

### Blood and bone marrow samples from breast cancer subjects

Bone marrow aspirates were obtained from patient's left and or right anterior or posterior iliac crests under anesthesia at the time of operation. A 10 or 20 cc syringe with a 16–18 gauge bone marrow aspirate needle was used to aspirate 3–6 ml of bone marrow into a syringe and then immediately transferred to a sterile EDTA vacutainer. Peripheral blood samples were obtained before surgery or following the induction of anesthesia. A total of 5–10 ml of blood was drawn from a peripheral vein into a sterile EDTA vacutainer. Blood and bone marrow samples were then shipped at room temperature to the Central Molecular Diagnostics Laboratory at the MUSC for immediate processing by Ficoll density gradient centrifugation (Ficoll-Paque Plus; Amersham Biosciences). All the specimens inside US arrived in 24 hours and international shipments arrived in 48 hours. One mL of bone marrow was used for Cytospin preparation and stained for ICC analysis. These bone marrow samples were evaluated by a cytopathologist for the presence of micrometastases using cytokeratin AE1/AE3. Please note that the specimen acquisition protocol was amended after the initiation of the MIMS trial and for that reason only a subset of patients was included in this analysis.

### Blood and bone marrow samples from control subjects without evidence of malignancy

In order to define baseline expression levels for the molecular markers used in this study, PBL and BM samples from control subjects were procured. Informed consent was obtained for BM aspiration from 49 patients undergoing orthopedic surgery at MUSC and for PBL drawn from 49 healthy volunteers. None of the control subjects had any history or clinical evidence of malignancy. Four to six ml of BM aspirate or 5–10 ml of PBL was transferred to an EDTA vacutainer and sent to the Central Molecular Diagnostics Laboratory to be processed by Ficoll density gradient centrifugation and analyzed by real-time RT-PCR.

### RNA isolation and cDNA synthesis

Buffy coats were obtained by Ficoll density gradient centrifugation, and total cellular RNA was isolated using a guanidinium thiocyanate-phenol-chloroform solution (RNA STAT-60™; TEL-TEST, Friendswood, TX). Briefly, cells were re-suspended in 1 ml of RNA STAT-60™. Total RNA was isolated as per the manufacturer's instructions with the exception that 1 μL of a 50 mg/mL solution of glycogen (Sigma, St. Louis, MO) was added to the aqueous phase prior to addition of isopropanol. Glycogen was used as a nucleic acid carrier to enhance RNA precipitation. The RNA pellet was dissolved in 50 μl of 1x RNA secure buffer (Ambion, Austin, TX). RNA was quantified by spectrophotometry at 260 nm. cDNA was made from 5 μg of total RNA using 200 U of M-MLV reverse transcriptase (Promega, Madison, WI) and 0.5 μg Oligo (dT)_12–16 _in a reaction volume of 20 μl (10 min at 70°C, 50 min at 42°C, 15 min at 70°C).

### Real-time RT-PCR

The real-time RT-PCR primers have been previously reported [31,36,37]: *mglo: *F 5'-GCCGTGTGAACCATGTGACTTT, R 5'-CCAAATGCGGCATCTTCAAA; *PDEF: *F 5'-AGTGCTCAAGGACATCGAGACG, R 5'-AGCCACTTCTGCACATTGCTG; *mam: *F 5'-CGGATGAAACTCTGAGCAATGT, R 5'-CTGCAGTTCTGTGAGCCAAAG; *CK19: *F 5'-CATGAAAGCTGCCTTGGAAGA, R 5'-TGATTCTGCCGCTCACTATCAG; *muc1: *F 5'-ACCATCCTATGAGCGAGTACC, R 5'-ACCATCCTATGAGCGAGTACC; *PIP: *F 5'-GCCAACAAAGCTCAGGACAAC, R 5'-GCAGTGACTTCGTCATTTGGAC; *EpCAM: *F 5'-CGCAGCTCAGGAAGAATGTG, R 5'-TGAAGTACACTGGCATTGACGA; *ErbB2*: F 5'-CTGGTGACACAGCTTATGCCCT, R 5'-ATCCCCTTGGCAATCTGCA. Analyses were performed on a PE Biosystems Gene Amp^® ^5700 Sequence Detection System (Foster City, CA). All reaction components were purchased from PE Biosystems. The standard reaction volume was 10 μl and contained 1X SYBR Green PCR Buffer; 3.5 mM MgCl_2_; 0.2 mM each of dATP, dCTP, and dGTP; 0.4 mM of dUTP; 0.25 U AmpliTaq Gold^®^; 0.1 U AmpErase^® ^UNG enzyme; 0.7 μl cDNA template; and 0.25 mM of both forward and reverse primer. The initial step of PCR was 2 min at 50°C for AmpErase^® ^UNG activation, followed by a 10-min hold at 95°C. Cycles (n = 40) consisted of a 15 sec denaturation step at 95°C, followed by a 1 min annealing/extension step at 60°C. The final step was a 60°C incubation for 1 min. All reactions were performed in triplicate. The cycle of threshold (C_t_) analysis was set at 0.5 relative fluorescence units.

### Primary data analysis

Real-time RT-PCR data were quantified as C_t _values that are inversely related to the amount of starting template: high C_t _values correlate with low levels of gene expression, whereas low C_t _values correlate with high levels of gene expression. Each gene was analyzed in triplicate. Results were normalized to an internal control reference gene, *β2-microglobin*, by subtracting the mean C_t _value of *β2-microglobin *from the mean C_t _value of each respective gene (ΔC_t _value). Samples for which C_t _values for *β*_2_*-microglobin *were equal or higher than 22 were considered to contain inadequate RNA and were excluded from the analysis. Approximately 10% of samples we rejected from the analysis based on this criterion. If the mean C_t _value for a gene of interest was higher or equal to 38, the gene expression was considered to be undetectable. In order to define baseline levels of gene expression and to define thresholds for marker positivity, 49 specimens of PBL and 49 specimens of BM obtained from patients with no evidence of malignancy were analyzed. To be consistent with the previous molecular analyses of lymph nodes, threshold values for each individual marker were set at three standard deviations from the mean ΔC_t _value in the control group. A subject was considered to be positive for the molecular analysis if at least one marker in the panel was above the defined threshold. Data from real-time RT-PCR analyses were compiled in a Microsoft Access database and submitted to the DCU at MUSC for statistical analyses. The molecular analysis was generated blinded to clinical outcome and patients' clinicopathologic data.

### Bone marrow cytopathology and cytokeratin ICC staining

Specimens were collected, washed in CytoLyt^® ^(Cytyc, Boston, MA) and then resuspended in PreservCyt^® ^(Cytyc). Two ThinPrep (TP) slides were prepared and stained with Papanicolaou stain, and one slide was used for immunocytochemistry (ICC). A monoclonal antibody for cytokeratin (AE1/AE3) was used in conjunction with an automated immunostaining system (DAKO Autostainer, DAKO Cytomation, Carpeteria, CA) and a Nexus immunohistochemistry slide staining apparatus (Ventana Medical Systems Inc, Tuscon, AZ). Immunostaining was performed with the avidin-biotin immunoperoxidase (ABC-peroxidase) method of Hsu et al [38]. Briefly, the slides were incubated with primary antibody for 30 minutes and then incubated with secondary biotinylated antibody for 4 minutes. To visualize the antibody, the TP was treated with diaminobenzidine (0.05%) in 0.05 M Tris-HCL buffer (pH 7.8) with 0.03% H_2_O_2 _for 6 minutes and then washed in H_2_O. TP was counterstained with hematoxylin, dehydrated, cleared in xylene, and mounted in Permount. The specimens were analyzed by a skilled cytopathologist.

### Statistical analysis

SAS Version 9.1 Software (SAS Institute Inc., SAS Campus Drive, Cary, North Carolina) was used for the analysis of pathological and molecular outcome. Chi-square analyses were conducted to explore the association between pre-defined baseline covariates that have been associated with pathological outcome in prior studies and PBL and BM RT-PCR positivity/negativity status. Pre-defined baseline covariates were tumor size, histological grade, estrogen receptor status, progesterone receptor status, her2neu status, and St. Gallen risk category (minimal/low risk: tumor size ≤ 1 cm, positive ER and/or PR status, grade I and age ≥ 35; intermediate risk: tumor size >1 or 2 cm, positive ER and/or PR status, and grade I; and high risk: lymph node positive, tumor size > 2 cm, negative ER and/or PR status, grade II or III, or age <35) [39]. Statistical significance was defined as p-values < 0.05.

## Results

### Demographic and clinicopathologic analysis

The distribution of the demographic and clinicopathologic characteristics in Table 1 indicate that the subset of patients with PBL analysis (n = 215) and the subset of patients with BM analysis (n = 177) are representative of the entire study group of 489 [33].

**Table 1:** Patient Demographic and Clinicopathologic Characteristics

**Characteristic**	**MIMS Trial study group (n = 489)**	**Peripheral blood (n = 215)**	**Bone marrow (n = 177)**
**Age**			
Mean (St. Dev.)	56.8 (11.4)	56.3 (10.9)	56.3 (10.8)
Range	26, 89	29, 84	30, 83
**Race**			
Caucasian	421 (86.1%)	180 (83.7%)	157 (88.7%)
Black	61 (12.5%)	31 (14.4%)	18 (10.2%)
Other	7 (1.5%)	4 (1.9%)	2 (1.1%)
**Primary Tumor**			
T1	334 (68.3%)	146 (67.9%)	117 (66.1%)
T2	145 (29.7%)	63 (29.3%)	52 (29.4%)
T3	10 (2.1%)	6 (2.8%)	8 (4.5%)
**Histologic Grade**			
I	133 (27.2%)	63 (29.3%)	53 (29.9%)
II	195 (39.9%)	89 (41.4%)	66 (37.3%)
III	136 (27.8%)	54 (25.1%)	49 (27.7%)
**Nodal Metastases (H&E)**			
N_0_	344 (70.4%)	150 (69.8%)	127 (71.8%)
N_1_	145 (29.7%)	64 (29.8%)	49 (27.7%)
**Nodal Metastases (PCR)**			
N_0_	251 (51.3%)	116 (54.0%)	94 (53.1%)
N_1_	238 (48.7%)	96 (44.7%)	80 (45.2%)
**Clinical Stage:**			
I	267 (54.6%)	120 (55.8%)	97 (54.8%)
IIA	138 (28.2%)	55 (25.6%)	47 (26.6%)
IIB	69 (14.1%)	33 (15.4%)	25 (14.1%)
IIIA	11 (2.3%)	7 (3.3%)	8 (4.5%)
IIIB	2 (0.4%)	0 (0%)	0 (0%)
IV	2 (0.4%)	0 (0%)	0 (0%)
**ER Status**			
ER-neg	107 (21.9%)	46 (21.4%)	42 (23.7%)
ER-pos	360 (73.6%)	154 (71.6%)	122 (68.9%)
**PR Status**			
PR-neg	156 (31.9%)	67 (31.2%)	58 (32.8%)
PR-pos	284 (58.1%)	120 (55.8%)	85 (48.0%)
**Her2neu Status**			
Her2neu-neg	218 (44.6%)	115 (53.5%)	91 (51.4%)
Her2neu-pos	89 (18.2%)	42 (19.5%)	30 (16.9%)
**Histologic Type**			
Infiltrating Ductal	394 (80.6%)	167 (77.7%)	140 (79.1%)
Infiltrating Lobular	44 (9.0%)	18 (8.4%)	16 (9.0%)
Other	50 (10.2%)	30 (14.0%)	21 (11.9%)
**Risk Category**			
Low Risk	53 (10.8%)	23 (10.7%)	16 (9.0%)
Intermediate Risk	50 (10.2%)	11 (5.1%)	13 (7.3%)
High Risk	386 (78.9%)	181 (84.2%)	148 (83.6%)

### Precise quantitation of gene-marker expression in normal control bone marrow and peripheral blood samples

**Figure 1:**
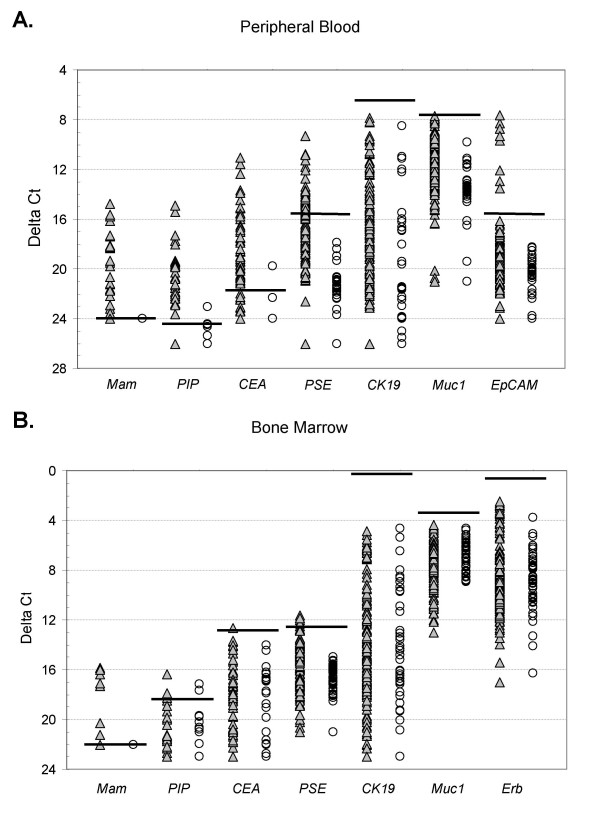
**Real-time RT-PCR analysis of cancer-associated gene expression in peripheral blood (A) and bone marrow (B) from breast cancer patients (filled triangle) and in normal control blood and bone marrow samples (empty circles)**. ΔC_t _values were obtained by subtracting the mean C_t _value of β2-microglobin from the mean C_t _value of each respective gene. C_t _values for each gene were determined from triplicate reactions. Horizontal lines indicate ΔCt threshold values (3 standard deviations from the mean). The ΔCt threshold for each gene are as follows: Peripheral blood: *mam *24.00, *PIP *24.19, *CEA *21.93, *PSE *15.28, *CK19 *6.32, *muc1 7.57*, *EpCAM *15.49; Bone marrow: *mam *22.00, *PIP *18.32, *CEA *12.64, *PSE *12.48, *CK19 *0.20, *muc1 *3.41, *ErbB2 *1.77.

We have previously shown that the majority of known breast cancer-associated genes have some background expression in normal lymph nodes [31,36,37]. For this study we selected seven breast cancer-associated genes [*mam, CEA, CK19, PIP, muc1, PSE, Erb *(BM only) and *EpCAM *(PBL only)] known to be over-expressed in metastatic breast cancer compared to control lymph nodes [31,36,37]. For this study, baseline gene expression was precisely quantitated in 49 normal PBL samples and 49 normal BM samples by real-time RT-PCR (Figure 1A and 1B; horizontal lines indicate the ΔCt thresholds). To obtain maximum specificity, a threshold value for marker positivity, i.e. abnormal expression was set at three standard deviations from the mean ΔC_t _value for each gene. Out of seven cancer-associated gene-markers used to detect tumor cells in PBL and BM, *CK19*, *muc1 *and *ErbB2 *were not informative due to the high expression in normal control samples.

### Real-time RT-PCR analysis of gene expression in peripheral blood of breast cancer patients

Using the five-marker gene-panel (*mam, PIP, CEA, PSE *and *EpCAM*) at the threshold of three standard deviations above the mean expression level in normal control samples for each gene, 136 (63%) patients out of 215 were positive for at least one marker. On an individual marker basis (Table 2), the most frequently over-expressed markers were *PSE *(58/215; 27.0%) and *CEA *(51/215; 23.7%) followed by *PIP *(36/215; 16.7%), *mam *(29/215; 13.5%) and *EpCAM *(7/215; 3.3%). Marker positivity in PBL demonstrated a statistically significant association with grade II-III (vs. grade I; p = 0.0083; Table 3). Out of 136 RT-PCR positive patients 97 patients (71%) were positive for one, 33 patients (24%) for two and six patients (4%) for three markers. Interestingly, over-expression of *PSE *gene had statistically significant association with ER-positive and PR-positive tumors (p = 0.0123 and p = 0.0134, respectively) and showed a trend towards pathology-negative nodal status (31% vs. 19%; Table 3). However, overexpression of *mam *gene had statistically significant association with high grade (p = 0.0315) and showed a trend towards ER-negative tumors (22% vs. 11%) and a high risk category (15% vs. 6%; Table 3). Interestingly, there was no association between marker positivity in PBL and either pathologic (H&E) status or molecular (multi-marker qRT-PCR) status of axillary lymph nodes.

**Table 2:** Positivity of cancer-associated genes in peripheral blood and bone marrow specimens

	**PBL_**N **_(n = 49)** **Ave. ΔCt (S.Dev.)**	**PBL_**BRCA **_(n = 215)** Positivity* (%)	**BM_**N **_(n = 49)** **Ave. ΔCt (S.Dev.)**	**BM_**BRCA **_(n = 177)** Positivity* (%)
***Mam***	24.00 (0.00)	29 (13.5%)	22.00 (0.00)	7 (4.0%)
***PIP***	25.84 (0.55)	36 (16.7%)	22.43 (1.37)	5 (2.8%)
***CEA***	23.88 (0.65)	51 (23.7%)	21.16 (2.84)	2 (1.1%)
***PSE***	22.60 (2.44)	58 (27.0%)	16.98 (1.50)	5 (2.8%)
***CK19***	21.41 (5.03)	0 (0%)	15.53 (5.11)	0 (0%)
***Muc1***	13.90 (2.11)	0 (0%)	6.77 (1.12)	0 (0%)
***ErbB2***	-	-	8.91 (2.38)	0 (0%)
***EpCAM***	21.40 (1.97)	7 (3.3%)	-	-

**Gene panel**	-	136 (63.3%)	-	19 (10.7%)

* ΔC_t _threshold was set 3 standard deviations from the mean in normal samples

**Table 3:** Association of molecular positivity in peripheral blood of breast cancer patients with traditional predictors of prognosis.

**Characteristic**	**5-gene panel (n = 136)**	***mam *(n = 29)**	***PSE *(n = 58)**
**Histologic Grade**			
I	31/63 (49.2%)	3/63 (4.8%)	15/63 (23.8%)
II-III	98/143 (68.5%)	22/143 (15.4%)	42/143 (29.4%)
	**P = 0.0083**	**P = 0.0315**	**P = 0.4110**
**Nodal Metastases (Path)**			
N_0_	97/150 (64.7%)	18/150 (12.0%)	46/150 (30.7%)
N_1_	37/64 (57.8%)	10/64 (15.6%)	12/64 (18.8%)
	**P = 0.3427**	**0.4715**	**P = 0.0726**
**ER Status**			
ER-neg	28/46 (60.9%)	10/46 (21.7%)	6/46 (13.0%)
ER-pos	98/154 (63.6%)	17/154 (11.0%)	49/154 (31.8%)
	**P = 0.7331**	**P = 0.0624**	**P = 0.0123**
**PR Status**			
PR-neg	40/67 (59.7%)	11/67 (16.4%)	12/67 (17.9%)
PR-pos	79/120 (65.8%)	16/120 (13.3%)	42/120 (35.0%)
	**P = 0.4033**	**P = 0.5650**	**P = 0.0134**
**Risk Category**			
Low/Intermediate Risk	20/34 (58.8%)	2/34 (5.9%)	9/34 (26.5%)
High Risk	116/181 (64.1%)	27/181 (14.9%)	49/181 (27.1%)
	**P = 0.5861**	P = 0.2698*	**P = 0.9564**

P-values obtained by Chi-square analysis or *Fisher's exact test

### Real-time RT-PCR analysis of cancer-associated gene expression in bone marrow

Using a four-marker gene-panel (*mam, PIP, CEA*, and *PSE*) at the threshold of three standard deviations above the mean expression level in normal control samples for each gene, 19 patients (11%) out of 177 were positive. All 19 were positive to one marker only. Marker positivity in bone marrow had no statistically significant association with any of the traditional prognostic indicators. Looking at individual markers separately (Table 2), the most frequently overexpressed marker was *mam *(7/177; 4.0%) followed by *PIP *(5/177; 2.8%), *PSE *(5/177; 2.8%) and *CEA *(2/177; 1.1%)

### Comparison of molecular analysis of blood and bone marrow

To determine whether there was an association between molecular analysis in PBL and molecular analysis in BM, we performed Chi-Square and Fisher's Exact test on 138 patients that had results from both PBL and from BM (Table 4). Comparison of the results using gene-panel data did not show statistically significant association, however, the results of *mam *and *PIP *gene expression in PBL had statistically significant association with the *mam *and *PIP *gene expression in BM (p = 2.5E-04 and p = 0.0188, respectively).

**Table 4:** Comparison between molecular analysis of peripheral blood (PBL) and molecular analysis of bone marrow (BM).

	**Gene panel**	***Mam***	***PIP***
**PBL(+)BM(+)**	13	5	3
**PBL(+)BM(-)**	5	2	2
**PBL(-)BM(+)**	69	11	16
**PBL(-)BM(-)**	51	120	117
**P-value**	**P = 0.2356**	P = 0.0002*	P = 0.0188*
**Concordance (%)**	64/138 (46.4%)	125/138 (90.6%)	120/138 (87.0%)

P-values obtained by Chi-square analysis or *Fisher's exact test

### Immunocytochemistry (ICC) versus RT-PCR in bone marrow

BM cytopathology assessment resulted in detection of no abnormal or suspicious cells. Eighty three BM samples were randomly selected for additional cytokeratin ICC staining. Five out of 83 (6%) samples were positive by ICC and two of these samples were also positive by RT-PCR (one positive for *mam *and other for *PIP*). Ten patients out of 83 (12%) that had inconclusive ICC results were all RT-PCR negative (Table 5). Although there was 84% agreement (excluding inconclusive ICC results) between 2 methodologies, this was mostly because of the concordance of dual negative findings. Overall there was no statistically significant association between ICC and PCR data (Chi-Square 0.1064; Fisher's exact test 0.1607; ICC inconclusive results excluded).

**Table 5:** Comparison between immunocytochemistry (ICC) and RT-PCR analysis in bone marrow.

	**Bone marrow**
**ICC(+)RT-PCR(+)**	2
**ICC(+)RT-PCR(-)**	3
**ICC(-)RT-PCR(+)**	9
**ICC(-)RT-PCR(-)**	59
**P-value**	P = 0.1607*
**Concordance (%)**	61/73 (84%)

*P-value obtained by Fisher's exact test

## Discussion

This paper describes molecular analyses of PBL and BM samples from a subgroup of breast cancer patients who were enrolled into a prospective multi-institutional study with the primary goal to establish the clinical relevance of micrometastatic disease detected by RT-PCR in pathology negative axillary lymph nodes. Our previous reports from this study strongly suggest that over-expression of cancer-associated gene-marker is a valid surrogate for occult micrometastatic breast cancer [33,34]. Using these gene markers [*mam, CEA, CK19, PIP, muc1, PSE, Erb *(BM only) and *EpCAM *(PBL only)] we analyzed 215 PBL samples and 177 BM samples from patients with T1-T3 primary breast cancer without clinical evidence of metastatic disease.

Using a predetermined rigorous threshold level (three standard deviations from the mean expression in normal PBL), 136 patients out of 215 (63.3%) had a positive signal in at least one cancer-associated marker in their PBL sample. According to the other studies, the incidence of CTC in PBL detected by RT-PCR ranged from 5% to 62% for one-marker analyses [13,15,16,19-24,26-29] and from 31% to 83% for analyses by multi-marker gene-panels [25-29]. The most frequently used markers were *CK19 *and *mam*. Our study, in contradiction, suggested that *CK19 *has high expression level in normal control samples and is therefore not reliable detector of CTC. Although the *CK19 *primers were designed to avoid the amplification of *CK19 *pseudogenes [40], we recognize that we cannot entirely exclude this possibility. In addition, we are aware of the limitations of using Ficoll density gradient cell separation methodology. Because of the low tumor cell burden in PBL and BM, the accuracy of tumor cell detection is greatly affected by the gene background expression levels. The genes like *CK19*, *muc1*, *PSE *and *EpCAM *that show significant background expression in normal samples, loose its accuracy in tumor cell detection when Ficoll density gradient cell separation methodology is used. In fact, in a separate publication we have demonstrated that using OncoQuick tumor cell enrichment method significantly reduces the background gene expression and therefore increases the sensitivity of tumor cell detection compared to the methodology employing Ficoll density gradient[27].

The *mam *gene on the other hand, because of its exquisite tissue specificity, did not show any expression in normal PBL. We observed a positive *mam *signal in 29 (13.5%) patients, which is comparable to studies by Roncella et al [20] and Benoy et al [13] who reported mam positivity in 12% (16/137) and 14% (16/116; M0) of patients, respectively. Other studies have showed *mam*-based CTC detection ranging from 41% to 62% [24,26,28,29].

Positivity thresholds for cancer-associated gene-expression in BM were also set at three standard deviations from the mean in normal BM. Based on this cut-off, 19 patients out of 177 (10.7%) were positive by RT-PCR. All 19 samples were positive for one cancer-associated marker. Additionally, in a subgroup of 83 BM samples analyzed by ICC, five (6%) resulted in a positive staining for cytokeratins. Two out of these five samples were also positive by RT-PCR (one for *mam *and another for *PIP*). Reports from other investigators on the incidence of DTC in BM detected by RT-PCR ranged from 12% to 53% [4,12-18] and as high as 80% [6] in metastatic disease. DTC detection by ICC for cytokeratins ranged from 13.2% to 62% (review by Braun et al [1]; [6]). In comparison to these reports the detection of DTC in our study appears to be relatively low. Although our study population contained mainly early stage breast cancer patients (55% in Stage I, 27% in Stage IIA,14% in Stage IIB and 5% in Stage IIIA), we also suspect that the limited volume of bone marrow (average of 3–4 ml) in combination of Ficoll density gradient methodology may not have been sufficient to achieve optimal sensitivity.

One of our goals in this study was to evaluate whether the expression of any individual gene was associated with poor prognostic indicators. Although the follow-up data for the breast cancer patients in this study is not yet available, we looked at the possible association of the detection of CTC and DTC with traditional clinicopathologic prognostic indicators employing Chi-Square and/or Fisher's exact tests. Among tumor size, histologic grade, ER-, PR-, Her2neu-status, lymph node status and high risk category, we observed a statistically significant association between marker positivity in PBL and histologic grade (grade II-III vs. grade I; p = 0.0083). There were no associations between marker positivity in PBL and pathologic (H&E) and/or molecular (multi-marker RT-PCR) status of axillary lymph nodes. Interestingly, overexpression of the mammaglobin gene alone had also statistically significant association with high grade (p = 0.0315) and showed a trend towards ER-negative tumors (22% vs. 11%) and a high risk category (15% vs. 6%), suggesting that *mam *gene may be a poor prognostic indicator (Table 3). Although we are not aware of other studies showing similar results on *mam*, there are reports of statistically significant association between *mam*-based CTC detection and tumor size [28], clinical stage [24,41], nodal status [42] and distant metastases [42-44] supporting the concept of *mam *gene being a poor prognostic indicator.

In our study, marker positivity in BM had no statistically significant association with any of the traditional prognostic indicators, however, the results of *mam *and *PIP *gene expression in PBL had statistically significant association with the *mam *and *PIP *gene expression in BM (p = 2.5E-04 and p = 0.0188, respectively; Table 4). We suggest that this result shows the close connection of PBL and BM compartments and that *mam *and *PIP *overexpression is not random but truly indicate the presence of tumor cells. Overall concordance between PBL and BM results were 90.6% for *mam *and 87.0% for *PIP*, which is mainly due to the concordance of double negative findings. Concordance for gene-panel was 46.4%. In comparison, Benoy et al demonstrated 68% of concordance between PBL and BM samples using *CK19 *and 75% concordance between PBL and BM samples using *mam *gene [13].

Clinical relevance of CTC in PBL and DTC in BM can only be studied with sufficient follow-up data. The most comprehensive study has been reported on detection of bone marrow micometastases published by Braun et al in *the New England Journal of Medicine *[1]. They performed a pooled analysis of a total of nine separate studies involving more than 4,500 breast cancer patients. Braun et al concluded that patients with BM micrometastases had poor overall survival (OS), breast-cancer-specific survival and poor disease-free survival (DFS) and distant-disease-free survival. A prospective, multi-center study by Cristofanilli et al used a new CellSearch System (Veridex) to determine if circulating tumor cells can predict survival in metastatic breast cancer. They tested 177 patients and found that patients with 5 or more tumor cells per 7.5 ml before the therapy and at the first follow-up visit had shorter median progression-free survival and OS compared to the patients with fewer than 5 circulating cells [30]. Benoy et al (CK19_PCR_, mam_PCR_) showed worse OS in patients with *CK19 *and *mam *expression in BM but not in PBL [13]. Median OS was reported to be shorter in patients with *CK*_ICC _positive cells in PBL according to Bauernhofer et al [9]. Detection of *CK19 *positive cells by RT-PCR in PBL in stage I and II was associated with reduced disease-free interval and OS [45].

## Conclusion

The interim results from this prospective clinical trial provides the first report of a statistically significant association between detection of *mam *mRNA in PBL and high grade breast tumors. Whether this result carries a clinical significance will be seen after the completion of the 5-year follow-up for this study.

## Abbreviations

PBL – peripheral blood. BM – bone marrow. CTC – circulating tumor cells. DTC – disseminated tumor cells. SLN – sentinel lymph node. ALN – axillary lymph node. RT-PCR – reverse transcription polymerase chain reaction. ICC – immunocytochemistry. *mam *– mammaglobin*. PIP *– prolactin-inducible protein*. CEA *– carcinoembryonic antigen*. PSE *– prostate-specific Ets factor. *CK19 *– cytokeratin 19*. Muc1 *– mucin 1*. EpCAM *– epithelial cell adhesion molecule. ErbB2 – Her2/neu*. Mglo *– beta-2-microglobulin. C_t _– cycle threshold.

## Competing interests

The author(s) declare that they have no competing interests.

## Authors' contributions

KM contributed to the study design and data interpretation, performed and supervised RNA extraction and real-time RT-PCR, drafted the manuscript. RHM contributed to the study design, performed the statistical analysis, contributed to data interpretation, drafted the manuscript. MBR contributed to the study design and data interpretation, performed RNA extraction and real-time RT-PCR. WEG contributed to the study design and data interpretation, drafted the manuscript. RH interpreted the bone marrow cytology and immunocytochemistry results. DHS provided normal bone marrow specimens. KC coordinated and carried out the protocols for obtaining normal peripheral blood and normal bone marrow specimens. MM contributed to the study design and data interpretation, supervised RNA extraction and real-time RT-PCR, drafted the manuscript. DJC designed the study, contributed to data interpretation, drafted the manuscript and served as a mentor for the entire project. All authors read and approved the final manuscript.

## Pre-publication history

The pre-publication history for this paper can be accessed here:


